# Stem Cells and Cartilage Development: Complexities of a Simple Tissue

**DOI:** 10.1002/stem.534

**Published:** 2010-09-29

**Authors:** Anthony P Hollander, Sally C Dickinson, Wael Kafienah

**Affiliations:** Department of Cellular & Molecular Medicine, University of BristolBristol, United Kingdom

**Keywords:** Chondrogenesis, Mesenchymal stem cells, Tissue regeneration, Adult stem cells

## Abstract

Cartilage is considered to be a simple tissue that should be easy to engineer because it is avascular and contains just one cell type, the chondrocyte. Despite this apparent simplicity, regenerating cartilage in a form that can function effectively after implantation in the joint has proven difficult. This may be because we have not fully appreciated the importance of different structural regions of articular cartilage or of understanding the origins of chondrocytes and how this cell population is maintained in the normal tissue. This review considers what is known about different regions of cartilage and the types of stem cells in articulating joints and emphasizes the potential importance of regeneration of the lamina splendens at the joint surface and calcified cartilage at the junction with bone for long-term survival of regenerated tissue in vivo. Stem Cells 2010;28:1992–1996

## INTRODUCTION

**Figure 1:**
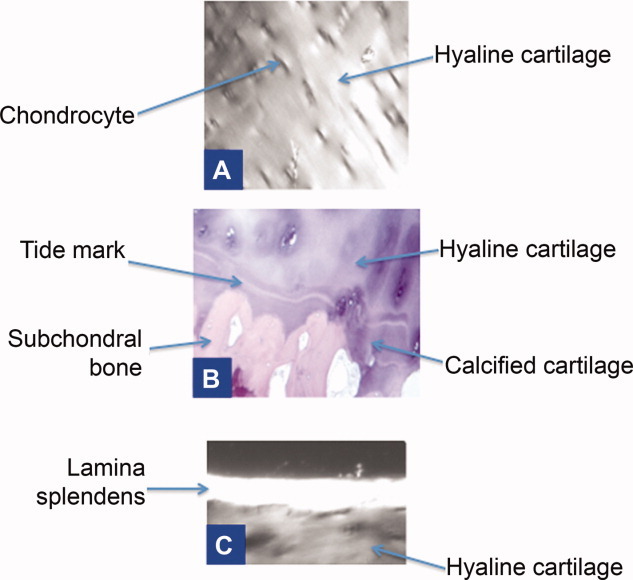
Histological appearance of different zones of articular cartilage. **(A):** The typical glassy appearance of hyaline cartilage under polarized light microscopy. **(B):** The calcified cartilage zone and tide mark at the cartilage–bone junction in hematoxylin and eosin stained sections. **(C):** The lamina splendens at the surface of articular cartilage under polarized light microscopy. All panels were viewed at ×10 magnification.

**Figure 2:**
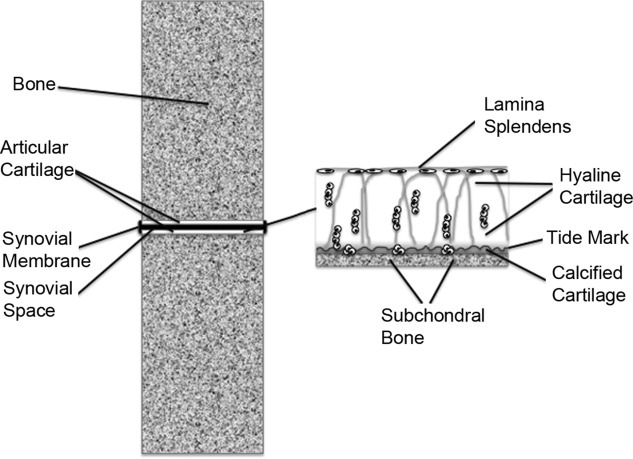
Diagram of the microstructure of articular cartilage found in the joints.

Damaged cartilage is considered by those of us who study it to be an ideal case for tissue engineering because it has no blood vessels or neurons, just chondrocytes. These enigmatic cells are obligate anaerobes and so are capable of withstanding the low oxygen tension of an avascular tissue. Cartilage is considered to be a simple structure, not only because of its single cell type but also because its extracellular matrix (ECM) is primarily accounted for by three molecules: water, type II collagen, and the large aggregating proteoglycan, aggrecan. This biochemical composition is uniquely suited to providing a combination of tensile strength with deformability, giving it mechanical properties that resemble those of a shock absorber [1], thereby dissipating forces across the bones, preventing them from fracturing during normal activity. The balance between mechanical stiffness and flexibility is itself the result of interaction between the thin type II collagen fibrils, giving tensile strength, within which are trapped molecules of aggrecan, which are highly negatively charged and so bind water avidly [1]. When unloaded, the water content of cartilage is about 70% of the wet weight. Under deforming load water flows out and when the load is reduced it flows back in, damping the effects of these forces. Damage to either the type II collagen or aggrecan may lead to loss of cartilage function [2,3]. If cartilage is to be engineered in the laboratory or repaired in vivo then the balance between collagen and proteoglycan must be restored to provide proper function. The capacity of newly implanted cartilage to survive without vascular in-growth combined with the relative simplicity of its ECM has made a compelling case for development of therapeutic strategies based on cartilage regeneration. The main structural feature of the ECM is hyaline cartilage, characterized by its glassy appearance when viewed under polarized light microscopy (Fig. 1A). This is also the major component of repair tissue in many patients [4]. The interface of hyaline cartilage and subchondral bone is bridged by calcified cartilage and this tissue is itself separated from the hyaline tissue by a proteoglycan-depleted tide-mark (Fig. 1B). The articular surface of intact cartilage consists of a distinct lamina splendens (Fig. 1C). Figure 2 shows the organizational relationship between these different regions of articular cartilage within the joint. The focus of most cartilage tissue engineering strategies has been on regenerating hyaline tissue and yet there is good reason to think that reconstruction of both the calcified cartilage and lamina splendens will be necessary for long-term survival of implants. This review focuses on the calcified cartilage and lamina splendens and suggests that different types of stem cell will be required to reproduce these structures in regenerating tissue.

## CURRENT APPROACHES TO CARTILAGE REGENERATION

Joint arthroplasty (the surgical implantation of artificial joints such as hips) is a highly successful intervention for osteoarthritis (OA) [5] but may lead to long-term pain [6] and aseptic loosening [7]. Tissue engineering could provide a step-change in OA treatment as it can allow healing of the natural joint, delaying the need for arthroplasty by several years or perhaps avoiding it altogether. Autologous chondrocyte implantation (ACI), a first generation tissue engineering approach, was first described by Brittberg et al. [8,9], with very good results for most patients treated over 9 years [9]. The technique involves expanding chondrocytes, taken from a biopsy of the patient's own cartilage, and injecting the cell suspension underneath a periosteal flap or collagen membrane, sutured over the lesion. It was a ground-breaking approach to cartilage repair that opened up the field of tissue engineering. However, it remains unclear if ACI offers a significant advantage over the simpler surgical technique of microfracture [10], which involves bleeding the subchondral bone at the lesion site to invoke clot formation. One explanation of this limited success of ACI may be that in most joint compartments of the knee focal cartilage lesions have a greater than 60% chance of increasing in size over a 2-year follow-up period [11]. Indeed, Poole et al. [12] have proposed that focal articular cartilage lesions represent the development of early OA. Regeneration of a mature, stable ECM can take as long as 1-2 years after ACI treatment [9], during which time the lesions may continue to increase in size. Therefore implantation of cartilage that has been pre-engineered in culture may prevent the growth in size of focal articular cartilage lesions in a way that is not possible with ACI. Indeed, chondrocytes precultured on scaffolds and implanted ectopically in nude mice generated better hyaline cartilage than cells implanted on scaffolds without preculture [13] and chondrocytes precultured on scaffolds for 14 days generated hyaline cartilage even when implanted in patients with preexisting early OA [4].

## STEM CELLS AND CARTILAGE REGENERATION

The next logical step is to engineer and implant cartilage with a more extensive ECM [14,15] and with an architecture that more closely resembles the natural tissue. Moreover, this mature implant will need to integrate with the surrounding host cartilage to survive and function [16]. Such an approach will require a sophisticated understanding of cartilage structure in development, maturity, and pathology. Scaling up tissue engineering therapies for the treatment of large numbers of patients will also ideally involve the use of allogeneic cells that can grow in vitro through multiple population doublings without senescing or transforming and can then differentiate into chondrocytes, produce appropriate matrix, and survive in vivo without immune rejection. However, the articular cartilage of joints sits on a rigid bony surface and is exposed to very high forces during normal daily activity [17]. The structure of any repair cartilage must therefore withstand these forces and implanted stem cells must respond appropriately to the loads since stiffness of the underlying tissue can have a marked impact on differentiation fate decisions [18].

Mesenchymal stem cells (MSCs) may be the ideal cell type for this purpose. Bone marrow-derived MSCs have been the most widely studied since their first isolation [19]. We have shown their potential for chondrogenic differentiation and tissue engineering [14,15]. They are hypoimmunogenic [20–23] and immunosuppressive [21,23] and so can create a zone of immune tolerance around the site in which they are implanted [20,21]. However, MSCs or MSC-like progenitors can be found within different tissues of the joint and these may have some advantages over the bonemarrow-derived cells in cartilage repair. Understanding the similarities and differences between these different stem cells may be crucial for effective articular cartilage regeneration.

If stem cells are to be the biological source of new cartilage then we must be able to control their differentiation so that they generate functional chondrocytes (the cells of cartilage) that can orchestrate the formation of an appropriate ECM. This has been the underlying principle of many attempts at cartilage repair [15,24].

However, creation of the osteochondral interface using stem cells will require a detailed understanding of those MSCs that are best able to differentiate to the hypertrophic chondrocyte phenotype.

## DEVELOPMENT AND REGENERATION OF CALCIFIED CARTILAGE

### Development of the Cartilage–Bone Interface

The cartilage adjacent to bone is calcified and like the calcified cartilage of the growth plate contains type X collagen. Histologically this area is demarcated by a tide mark in hematoxylin and eosin-stained sections, running parallel to the bone surface (Fig. 1B) [25]. The elastic modulus of calcified cartilage is in the MPa range, compared with kPa values for hyaline cartilage and GPa for the underlying bone [26,27]. These observations indicate the importance of calcified tissue as a transition between cartilage and bone. Engineered implants that have failed to recreate this transition may fail under mechanical load.

Long bones start growing in the early stages of fetal development, continuing in neonates, and through to skeletal maturity. The mechanism of bone formation is endochondral ossification. An epiphyseal cartilage template is gradually ossified at the primary and then secondary centers of ossification. Chondrocytes undergo terminal differentiation to hypertrophic chondrocytes that are positive for alkaline phosphatase and type X collagen as well as for type II collagen and have the capacity to mineralise their ECM [1]. As the hypertrophic epiphyseal cartilage is calcified, a process of vascular invasion leads to repopulation of the cartilage with osteoblasts that remodel the calcified cartilage into bone [1]. This process creates a continuum from cartilage to bone. After the growth plates have closed and bone growth has ceased, the cartilage remains as a thin layer at the end of the bone (the articulating surface) and the cartilage–bone interface is characterized by calcified cartilage that differs from hypertrophic cartilage in the growth plate because it is not normally invaded by blood vessels and therefore it does not remodel to bone. The chondrocytes in and adjacent to the calcified cartilage produce both type X collagen [28] and alkaline phosphatase [29].

### Engineering the Cartilage–Bone Interface

Tissue engineering may be able to generate calcified cartilage at the hyaline cartilage–subchondral bone interface through a combination of appropriate use of biomaterial scaffolds, careful selection of specific progenitor cells, and regulation of biomechanical signaling in vitro either through biomaterial stiffness or through loading regimes in purpose-designed bioreactors [18,30,31]. However, this would create a further problem of how to integrate engineered calcified cartilage with natural subchondral bone. Therefore, a more realistic approach may be to rely on the mechanical and growth factor signals within the in vivo implantation site as the most important regulatory factors, driving maturation of the tissue in situ [32]. Bone marrow-derived MSCs are thought to have an inherent tendency to differentiate to hypertrophic chondrocytes; however, recent in vivo studies [33,34] have suggested that on implantation into articular cartilage they only terminally differentiate in the deep zone adjacent to bone, whereas in more superficial zones they are arrested in a prehypertrophic state by the local production of parathyroid hormone-related protein (PTHrP). Therefore, use of bone marrow-derived MSCs rather than chondrocytes may provide a simple solution to the problem of creating a zone of calcified cartilage. On the other hand, loss of the surface zone of articular cartilage in OA may mean there is no PTHrP production leading to aberrant calcification throughout the implant side. Therefore, an understanding of the stem cell properties and the nature of the implant site will be critical for ensuring an effective tissue engineering outcome.

## DEVELOPMENT AND REGENERATION OF THE SURFACE OF ARTICULATING CARTILAGE

### Development of the Surface Zone

At the very surface of articulating cartilage is the lamina splendens in which collagen fibrils run parallel to the surface of articulation [35]. This surface zone layer is likely to play a key role in maintaining the mechanical response of articular cartilage to load [36]. It is the first region of cartilage to degrade in OA [37,38] and there is no evidence that it is regenerated when chondrocytes are implanted into lesion sites [4,39]. This is a significant limitation that, unless resolved, may lead to the ultimate failure of any articular cartilage implant as a result of the shear forces in the joint. For this reason, it is critical to understand the role of the surface zone of cartilage in development, joint function, and pathology. It is also essential to consider the importance of repair strategies that can result in regeneration of the surface zone.

Hayes et al. [40] explored the shift during cartilage development from a relatively simple isotropic tissue with a high cell density and homogeneous distribution of collagen fibrils to an anisotropic tissue with a low density of chondrocytes growing in vertical columns and a unique arrangement of collagen fibrils. They studied these changes in the marsupial “Monodelphis Domestica” because of its short gestation and primitive stage of development at birth. They concluded that the tissue develops through appositional growth from the articular surface with a gradual fall in cell density in the surface zone as the dividing progenitor cells undergo asymmetric division, giving rise to transit-amplifying cells. The same team went on to isolate progenitor cells from the surface zone of 7-day-old bovine calf articular cartilage [41,42]. They were isolated based on their ability to bind well to fibronectin and to form colonies with greater efficiency than chondrocytes from elsewhere (or compared with those cells not binding to fibronectin). They characterized the cells as being positive for notch 1. Lineage-labeled cells were able to differentiate into musculoskeletal tissues when implanted into chick eggs. They can continue to grow in vitro for up to 50 population doublings (approximately 17 passages) before showing signs of senescence. The growth kinetics of these cells are typically very rapid at first but then slowing down. In contrast, the chondrocyte population in general shows linear growth kinetics irrespective of time in culture. The chondrogenic potential of the surface zone progenitor cells is maintained right through to late passage, until senescence.

The origin of these surface zone progenitor cells and the extent to which they persist into maturity remains unknown. These issues were explored further by Karlsson et al. [43] who investigated stem cell populations throughout the joints of skeletally mature 3-month-old rabbits. In particular, they identified the zone of Ranvier as a potential MSC niche in the joint. This zone is located at the edge of the growth plate of long bones in rabbits. These cells are positive for Jagged-1 and Stro-1. Importantly, stem cells in the articular cartilage itself were not found just at the surface but throughout the cartilage at relatively low density. This contrasts with the previous findings of surface zone chondroprogenitors in 7-day-old calves [41,42]. Karlsson et al. conclude that the articular cartilage chondroprogenitor cells are derived from migration of mesenchymal cells out of the zone of Ranvier niche. They hypothesize that in early development these cells may accumulate in the surface zone and drive the process of appositional growth of cartilage but with maturity they become dissipated throughout the cartilage. These observations may explain why lesions in the articular surface of cartilage may heal spontaneously in immature animals [44] whereas in adult animals there is no evidence of spontaneous healing [45].

Thus, the surface zone of articular cartilage is a critical component of the mature tissue because its collagen fibrils are oriented parallel to the plane of the tissue surface and so endow it with resistance to shear forces in the joint. It is also a critical component of the immature tissue because it drives appositional growth and may allow spontaneous healing when there is fibrillation at the surface [41–43]. It follows that tissue-engineered cartilage implants that do not have a lamina splendens will not function mechanically in the same way as the natural tissue because they will lack resistance to shear forces. Establishing a zone of type II collagen fibrils in the appropriate orientation at the surface should be a key aim of any cartilage engineering strategy. Furthermore, the long-term survival of engineered implants in the hostile environment of an osteoarthritic joint may depend on the capacity to establish a population of progenitor cells within the surface zone that can drive a repair process when damage has accrued. It is important to note that at least one study [46] has shown a lack of type II collagen in this zone. Instead the parallel bundles of collagen fibrils were found to be composed of collagen types I and III. Therefore, the chondroprogenitors in this zone may have a characteristic collagen synthesis phenotype that is different to cells found elsewhere in articular cartilage.

### Engineering the Surface of Cartilage

Enriching the surface of engineered implants with mesenchymal cells derived from the synovial membrane may be a way of reconstructing the lamina splendens. De Bari isolated mesenchymal cells from the synovial tissue of adult humans and showed them to have the capacity to undergo chondrogenic, osteogenic, adipogenic, and myogenic differentiation [47,48]. He went on to isolate clones of synovial membrane progenitor cells that were able to grow for 25–50 population doublings before undergoing senescence. These clones were negative for markers of hematopoetic stem cells (CD45) and endothelial cells (CD31) but positive (with variable expression) for MSC markers CD13, CD105, CD73, CD166, SSEA4, CD81, and CD90. Thus, the synovium-derived cells are compatible with an MSC phenotype. All the clones were chondrogenic in pellet cultures, even at late passage, as well as osteogenic, whereas adipogenesis was more variable.

Lee et al. made the interesting and potentially important observation that after chondrogenic differentiation, synovial membrane-derived progenitors secrete lubricin, also known as “surface zone protein” [49]. This glycoprotein protects the articular surface-adherent proteins from cell infiltration as well as providing boundary lubrication that may be critical for mechanical function of the joint [50]. Lubricin is also found at the surface of other joint tissues such as ligaments [50]. The synovial membrane synthesizes synovial fluid that bathes the surface of all joint structures and so the synovial membrane-fluid pathway may provide a route for synovial MSCs to migrate between different tissues of the joint, differentiate, and (at the surface) secrete lubricin. Whether these cells also participate in the maintenance of the lamina splendens is not known. However, the concept of seeding synovial MSCs onto the surface of engineered cartilage to create a lubricin-rich zone is attractive and may at least enhance the mechanical function of the biological implants.

## SUMMARY

Loss of the surface zone early in OA may be devastating because it will remove the main driver of the appositional growth of cartilage. Failure of repair of the lamina splendens may reflect a failure of this niche to function normally in the injured adult joint, leading ultimately to cartilage erosion and loss of joint function. Tissue engineering solutions will most likely require a recreation of the surface zone if the implants are to survive without rapid degradation. Similarly, recreation of the calcified cartilage that interfaces the osteochondral junction is likely to be essential for the avoidance of delamination as a result of shear forces focusing stresses at this site. Cartilage is a simple tissue that is full of complexity and this must be reflected in our stem cell and tissue engineering strategies.

## DISCLOSURE OF POTENTIAL CONFLICTS OF INTEREST

The authors indicate no potential conflicts of interest.
